# Telomerase reverse transcriptase and telomeric-repeat binding factor protein 1 as regulators of telomerase activity in pancreatic cancer cells

**DOI:** 10.1054/bjoc.2001.1954

**Published:** 2001-09

**Authors:** T Yajima, A Yagihashi, H Kameshima, D Kobayashi, K Hirata, N Watanabe

**Affiliations:** Department of Clinical Laboratory Medicine, Sapporo Medical University School of Medicine, Sapporo, Japan; Department of Surgery, Sapporo Medical University School of Medicine, Sapporo, Japan

**Keywords:** telomerase, hTERT, hTRF1, TRF, X-irradiation, pancreatic cancer cell

## Abstract

Telomerase adds hexameric repeats of 5′-TTAGGG-3′ termed telomeres to ends of chromosomal DNA. This enzyme has been implicated in cellular immortalization and cellular senescence. Recently, a number of relevant genes have been cloned, including these encoding three major components of human telomerase: human telomerase RNA component (hTR), human telomerase reverse transcriptase (hTERT), and telomerase-associated protein-1 (TEP1). Also important are genes encoding human telomeric-repeat binding factor protein (TRF) 1 and 2. To clarify mechanisms regulating telomerase activity, we studied telomerase activity, the telomeric restriction fragment (TRF) length and gene expression of these telomerase components and the telomeric-repeat binding factor proteins in sequential observation following X-irradiation of cultured pancreatic cancer cells. We previously reported that PANC-1 cells are better able to tolerate thermal stress, antineoplastic drugs, and exposure to tumour necrosis factor than MIAPaCa-2 cells. MIAPaCa-2 and PANC-1 cells were exposed to X-irradiation, their telomerase activity was increased at 2 days and then decreased gradually. Of the three telomerase components, only hTERT mRNA expression showed parallel changes. TRF length was stable just before and after X-irradiation. Among binding factor proteins, TRF1 mRNA showed reciprocal changes possibly directed toward maintaining a stable telomere length. In this study, our results demonstrate that not only hTERT but also TRF1 are important regulator of telomerase activity. © 2001 Cancer Research Campaign http://www.bjcancer.com


*British Journal of Cancer* (2001) **85**(5), 752–757
© 2001 Cancer Research Campaign
doi: 10.1054/ bjoc.2001.1954, available online at http://www.idealibrary.com on
http://www.bjcancer.com

### Telomerase reverse transcriptase and telomeric-repeat binding factor protein 1 as regulators of telomerase activity in pancreatic cancer cells

**T Yajima[1], A Yagihashi[1], H Kameshima[1], D Kobayashi[1], K Hirata[2] and N Watanabe[1]**

[1]Department of Clinical Laboratory Medicine; [2]Department of Surgery, Sapporo Medical University School of Medicine, Sapporo, Japan

**Summary** Telomerase adds hexameric repeats of 5′-TTAGGG-3′ termed telomeres to ends of chromosomal DNA. This enzyme has been implicated in cellular immortalization and cellular senescence. Recently, a number of relevant genes have been cloned, including these encoding three major components of human telomerase: human telomerase RNA component (hTR), human telomerase reverse transcriptase (hTERT), and telomerase-associated protein-1 (TEP1). Also important are genes encoding human telomeric-repeat binding factor protein (TRF) 1 and 2. To clarify mechanisms regulating telomerase activity, we studied telomerase activity, the telomeric restriction fragment (TRF) length and gene expression of these telomerase components and the telomeric-repeat binding factor proteins in sequential observation following X-irradiation of cultured pancreatic cancer cells. We previously reported that PANC-1 cells are better able to tolerate thermal stress, antineoplastic drugs, and exposure to tumour necrosis factor than MIAPaCa-2 cells. MIAPaCa-2 and PANC-1 cells were exposed to X-irradiation, their telomerase activity was increased at 2 days and then decreased gradually. Of the three telomerase components, only hTERT mRNA expression showed parallel changes. TRF length was stable just before and after X-irradiation. Among binding factor proteins, TRF1 mRNA showed reciprocal changes possibly directed toward maintaining a stable telomere length. In this study, our results demonstrate that not only hTERT but also TRF1 are important regulator of telomerase activity. © 2001 Cancer Research Campaign http://www.bjcancer.com

**Keywords**: telomerase; hTERT; hTRF1; TRF; X-irradiation; pancreatic cancer cell

Telomerase is an enzyme that replaces repetitive $(TTAGGG)_n$ sequences on ends of chromosomes that otherwise are lost with successive cell divisions (Grieder et al, 1985). As a result telomerase activity is linked closely to attainment of cellular immortality, a step in carcinogenesis, while lack of such activity contributes to cellular senescence (Kim et al, 1994; Shay et al, 1997). Recently, genes encoding three major components of human telomerase have been cloned, specifically human telomerase RNA component (hTR) (Feng et al, 1995), human telomerase reverse transcriptase (hTERT) (Nakamura et al, 1997; Meyerson et al, 1997), and telomerase-associated protein-1 (TEP1) (Harrington et al, 1997; Nakayama et al, 1997). Other recently cloned sequences code for human telomeric-repeat binding proteins, such as human telomeric-repeat binding factor protein (TRF) 1 and 2 (Chong et al, 1995; Broccoli et al, 1997).

Expression of these genes can now be studied and could be therapeutically altered. However, details of mechanisms regulating telomerase activity are still poorly understood, and, as a result, the specific components or binding proteins that might represent suitable targets for cancer gene therapy have not been identified. Since expression levels of these genes have been studied by varying methods of Northern blotting or reverse transcription-polymerase chain reaction (RT-PCR) that make comparisons difficult (Feng et al, 1995; Nakamura et al, 1997; Meyerson et al, 1997; Harrington et al, 1997; Nakayama et al, 1997; Broccoli et al, 1997; Nakayama et al, 1998; Ito et al, 1998; Takakura et al, 1998; Kyo et al, 1999), we performed novel quantitative assays using a TaqMan RT-PCR for the telomerase components hTR, hTERT and TEP1 as well as for TRF1 and TRF2 (Yajima et al, 1998; Yajima et al, 2000).

Radiation induces cellular damage at DNA level. A possible role of telomerase activity in radiation induced cellular damage is to maintain the integrity of the genome including telomeric region. Low dose of X-irradiation induces up-regulation of telomerase activity (Hyeon et al, 1998). However, little is known about the effect of X-irradiation to the transcription of these telomerase components and TRFs.

To clarify mechanisms regulating telomerase activity, we studied telomerase activity, telomere length and gene expression of these telomerase components and the TRFs in sequential observation following X-irradiation of cultured cells.

*Received 9 March 2000*
*Revised 1 June 2001*
*Accepted 4 June 2001*

*Correspondence to:* N Watanabe, Department of Clinical Laboratory Medicine, Sapporo Medical University School of Medicine, South-1, West-16, Chuo-ku, Sapporo 060-8543, Japan

### MATERIALS AND METHODS

### Cells

The human pancreatic cancer cell lines PANC-1, and MIA PaCa-2, were obtained from the American Type Culture Collection (ATCC; Manassas, VA). PANC-1 cells are better able to tolerate thermal stress, antineoplastic drugs, and exposure to tumour necrosis factor than MIAPaCa-2 cells, as reported previously (Watanabe et al, 1996; Watanabe et al, 1997). We therefore, used these two different cell lines in the present study. PANC-1 cells were cultured in RPMI 1640 medium supplemented with 10%

BJOC 01-1954 752-757 20/8/01 3:18 pm Page 753

heat-inactivated FCS. MIAPaCa-2 cells were cultured in Dulbecco's modified Eagle's medium (Nipro) supplemented with 10% heat-inactivated FCS and 2.5% heat-inactivated horse serum.

### Irradiation

PANC-1 or MIAPaCa-2 cells ($5 \times 10^6$) were seeded into tissue culture dishes 100 mm in diameter (Falcon, Oxnard, CA) containing 10 ml of culture medium. After a 6-h incubation, the culture medium was changed and cells were X-irradiated with a dose of 10, 20, or 30 Gy. X-irradiation was carried out at room temperature using an MBR-1520A-TW device (20 mA, 150 kV; Hitachi Medical, Tokyo, Japan) at a dose rate of 2.089 Gy/min. Cells were harvested for either quantitative RT-PCR assay or assay for telomerase activity preceding irradiation or after and 1, 2, 4, 6, or 8 days.

### Telomerase assay

Cells were trypsinized and collected as pellets after centrifugation at $1000 \times g$ for 5 min at 4˚C. The pellets then were washed and lysed as described previously (Kim et al, 1994). After incubation on ice for 30 min, lysates were centrifuged at $16\,000 \times g$ for 30 min at 4˚C. Supernatants were collected and their protein concentrations were measured by a Gene Quant DNA/RNA Calculator (Pharmacia, Camblidge, UK); lysates contained 0.06 µg of cellular protein. Telomerase activity was measured using a PCR-based TRAP-eze Telomerase Detection Kit (Oncor, Gaithersburg, MD) according to the manufacturer's instructions. Briefly, for end-labeling of TS primer, 10 µl of TS primer (5′-AATCCGTCGAGCAGAGTT-3′) was added to 10 µl reaction mixture including 10 µCi of [$\gamma$-$^{32}$P] ATP (3000 Ci/mmol; Amersham, Cambridge, UK), 2 µl of $10 \times$ kinase buffer (Takara, Kyoto, Japan), 5 U of T4 polynucleotide kinase, and 5 µl of distilled (d) $H_2O$. The mixture was incubated for 20 min at 37˚C and then for 5 min at 85˚C. One microlitre of this primer mixture was added to a reaction mixture containing 5 µl of $10 \times$ TRAP buffer, 1 µl of $50 \times$ dNTPs, 2 µl of TS end-labeling primer, 1 µl of TRAP primer mix, 2 U of Taq DNA polymerase (Takara), 38.6 µl of $dH_2O$, and 2 µl of CHAPS extract (total volume, 50 µL). Each TRAP reaction mixture was incubated at 30˚C for 30 min followed by 27 cycles of 94˚C for 30 s and 60˚C for 30 s in a thermal cycler (model 9600; Perkin-Elmer, Foster City, CA). Fifteen microlitres of the PCR product was electrophoresed in $0.5 \times$ Tris-borate EDTA buffer on 12.5% polyacrylamide nondenaturing gels. Gels were dried and processed for autoradiography, exposing sensitive New A film (Konica, Tokyo, Japan) at –80˚C for 3 h. Signal intensity on exposed films was measured using Personal Densitometer model SI (Molecular Dynamics, Sunnyvale, CA). The experimental sample was incubated at 85˚C for 10 min prior to the TRAP assay to inactivate telomerase and serve as a negative control, and a cell extract of known telomerase content provided in the kit served as a positive control.

Semiquantitative analysis to estimate relative telomerase activity was accomplished by performing the TRAP assay with a TSR8 control template provided in the kit in place of sample extract. Telomerase activity was calculated as unit indicated total product generated (TPG) using a formula as described previously (Kim et al, 1997): $TPG = \{[(x - x_0)/c]/[(r - r_0)/c_R]\} \times 100$, where telomerase products from non-heat-treated sample extract were x, telomerase products from a heat-treated sample extract were $x_0$, a non-heat-treated sample extract as an internal control was c, telomerase product TSR8 quantification control was r, telomerase products from the lysis buffer only was $r_0$, and the internal control for TSR8 quantification was $c_R$.

### Southern blotting and terminal restriction fragment (TRF) length analysis

Genomic DNA was extracted from cells by using SepaGene (Sanko Pure Chemical). 10 µg of DNA was digested with *Hin*fI (Sigma) at 37˚C for 4 h and electrophoresed on 1% agarose gels. The digested DNAs were then blotted on to nylon membranes (Boehringer, Mannheim, Germany) and hybridized with $^{32}$P-labelled $(TTAGGG)_4$ probe. The hybridized blots were washed and then autoradiographed as reported previously (Hiyama et al, 1996). We estimated the mean length of terminal restriction fragments at the peak position of hybridization signal using Personal Densitometer model SI.

### Quantitative RT-PCR assays for hTR, hTERT mRNA, TEP1 mRNA, TRF1 mRNA, and TRF2 mRNA

Cells were trypsinized and the cell pellets were collected by centrifugation at $1000 \times g$ for 5 min at 4˚C. SepaGene RV-R (Sanko Pure Chemical, Tokyo, Japan) was used to extract total RNA from cells, and this extract was assayed for RNA with Gene Quant DNA/RNA Calculator (Pharmacia). Contaminating chromosomal DNA was digested with DNAse I (GIBCO-BRL, Gaithersburg, MD) according to the manufacturer's instructions. For quantitative RT-PCR, fluorescent hybridization probes, TaqMan PCR Core Reagents Kit with AmpliTaq Gold (Perkin-Elmer) were used with the ABI Prism 7700 Sequence Detection System (Perkin-Elmer). Expression of hTR, hTERT mRNA and TEP1 mRNA were quantified by methods previously reported (Yajima et al, 1998; Yajima et al, 2000). Expression of TRF1 and TRF2 mRNA was quantified using a method similar to that for hTR, hTERT mRNA and TEP1 mRNA (Yajima et al, 1998; Yajima et al, 2000). Primers and TaqMan probes for TRF1 and TRF2 mRNA were as follows. Sequences of the forward primer for TRF1 mRNA were 5′-GCAACAGCGCAGAGGCTATTATT-3′ and the reverse primer, 5′-AGGGCTGATTCCAAGGGTGTAA-3′; the sequence of the TaqMan probe was 5′-TCCAGTCTAACAGCTTGCCAGTTGA-GAACG-3′. Sequences of the forward primer for TRF2 mRNA were 5′-AAACGAAAGTTCAGCCCCG-3′ and the reverse primer, 5′-TCCTCCAAGACCAATCTGCTTA-3′; the sequence of the TaqMan probe was 5′-CAGCCCAAGAACAAGCGCATGACA-3′. Conditions of one-step RT-PCR were as follows: 30 min at 48˚C (stage 1, reverse transcription), 10 min at 95˚C (stage 2, RT inactivation and AmpliTaq Gold activation), and then 40 cycles of amplification for 15 s at 95˚C and 1 min at 60˚C (stage 3, PCR). Data for hTR, hTERT mRNA, TEP1 mRNA, TRF1 mRNA, and TRF2 mRNA were normalized to data for glyceraldehyde-3-phosphate dehydrogenase (GAPDH).

### RESULTS

### Changes in cell number; telomerase activity, and gene expression for telomerase components in MIAPaCa-2 and PANC-1 cells after X-irradiation

To manipulate telomerase activity, MIAPaCa-2 and PANC-1 cells were X-irradiated with dose of 10, 20, or 30 Gy. Figure 1 shows cell numbers just before and at 1, 2, 4, 6, and 8 days following X-irradiation. Under all conditions, PANC-1 cells were more

*© 2001 Cancer Research Campaign* 

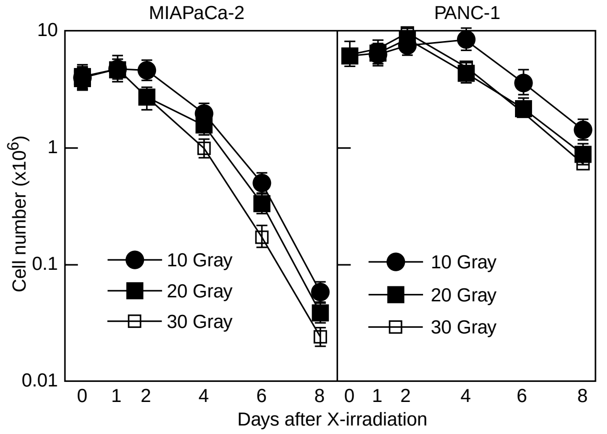

**Figure 1** Cell numbers surviving over time after X-irradiation (10, 20, or 30 Gy). PANC-1 cells were more resistant to irradiation than MIAPaCa-2 cells resistant to X-irradiation than MIAPaCa-2 cells. Time courses of telomerase activity and gene expression for the telomerase components were examined after 20 Gy irradiation. Two days after irradiation, telomerase activity of MIAPaCa-2 and PANC-1 cells respectively increased to 134.1% and 160.2% of pre-irradiation activity, and then gradually fell to 13.5% and 10.4% of baseline by 8 days after irradiation (Figure 2). As for gene expression of telomerase component genes, the level of hTERT mRNA expression peaked 2 days after irradiation, and later decreased gradually (Figure 3), a sequence similar to that for changes in telomerase activity. In contrast, hTRand TEP1 mRNA expression

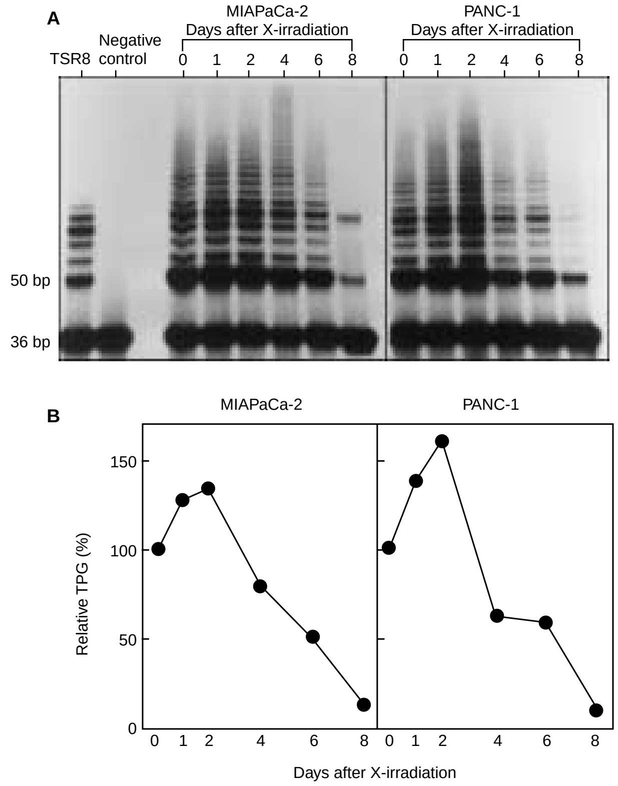

**Figure 2** Telomerase activity over time after 20 Gy of X-irradiation to MIAPaCa-2 and PANC-1 cells. Telomerase activity was quantified by units of total product generated (TPG), and is expressed as a relative percentage of TPG measured in cells just before X-irradiation

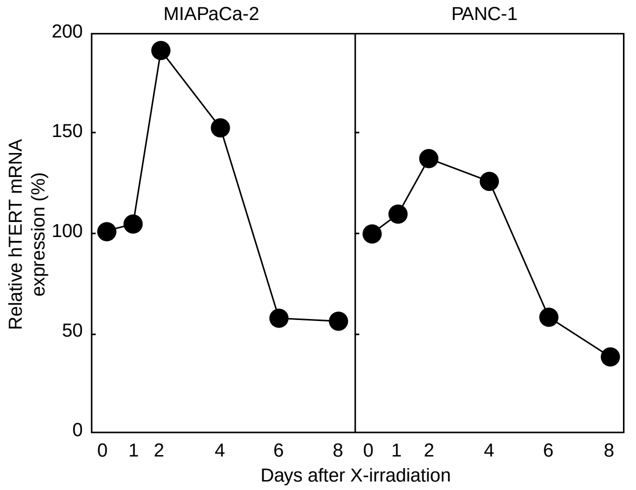

**Figure 3** Expression of hTERT mRNA over time after 20 Gy of X-irradiation to MIAPaCa-2 and PANC-1 cells. Expression of hTERT mRNA was measured by a quantitative TaqMan RT-PCR assay, and is stated relative to that of glyceraldehyde-3-phosphate dehydrogenase. Relative hTERT mRNA expression is calculated as a relative percentage of cells just before X-irradiation. hTERT, human telomerase reverse transcriptase increased gradually after X-irradiation (Figures 4 and 5). These results suggest that expression of hTERT may play important role for regulation of telomerase activity. We examined time courses of telomerase activity after 10 and 30 Gyirradiation. After 10 Gy irradiation, telomerase activity did not decrease to less than 50% of that in cells just before X-irradiation, and after 30 Gy, the cell number at 8 days was not enough to examine telomerase activity, expression of its component and TRF length.

### Telomeric restriction fragment (TRF) length in MIAPaCa-2 and PANC-1 cells after X-irradiation

To elucidate whether the change of telomeric length occurs after X-irradiation, the telomeric restriction fragment (TRF) which is an indicator of telomeric length was examined by Southern

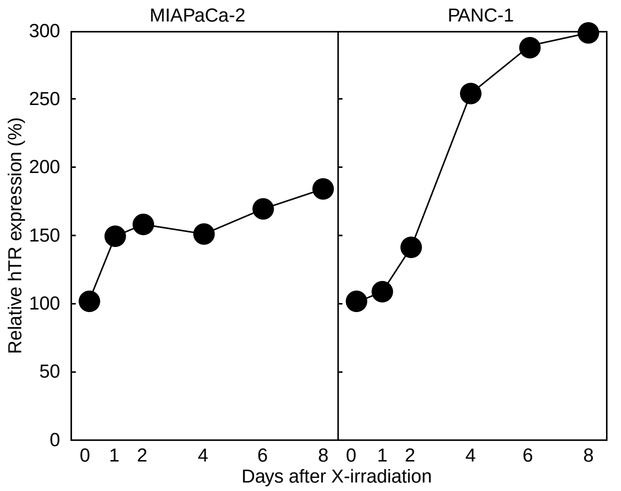

**Figure 4** Expression of hTR over time after 20 Gy of X-irradiation to MIAPaCa-2 and PANC-1 cells. Expression of hTR was quantified by a quantitative TaqMan RT-PCR assay, and is stated relative to that of glyceraldehyde-3-phosphate dehydrogenase. Relative hTR expression is calculated as a relative percentage of cells just before X-irradiation. hTR, human telomerase RNA component

 *© 2001 Cancer Research Campaign*

BJOC 01-1954 752-757 20/8/01 3:18 pm Page 755

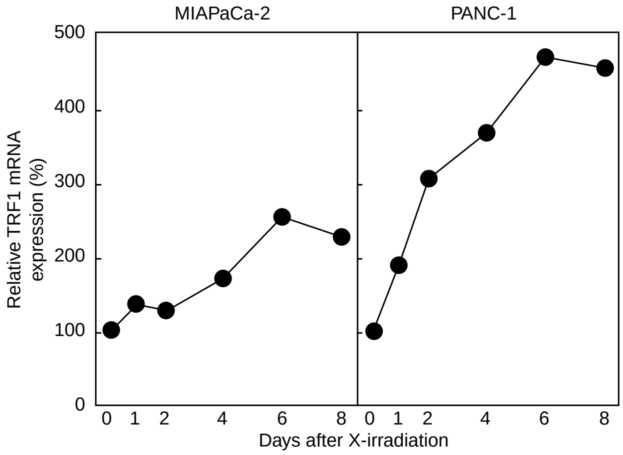

**Figure 5** Expression of TEP1 mRNA over time after 20 Gy of X-irradiation to MIAPaCa-2 and PANC-1 cells was measured by a quantitative TaqMan RT-PCR assay. Expression is stated relative to that of glyceraldehyde-3-phosphate dehydrogenase, and relative TEP1 mRNA expression is calculated as a relative percentage of cells just before X-irradiation. TEP1, telomerase-associated protein-1

hybridization assay. Figure 6 shows TRF length just before and at 1, 2, 4, 6, and 8 days following X-irradiation. TRF length was stable between 2.7 and 2.8 kb, 2.5 and 2.6 kb in MIAPaCa-2 and PANC-1 cells, respectively.

### Telomerase activity and gene expression for telomeric-repeat binding factors in MIAPaCa-2 and PANC-1 cells after X-irradiation

To clarify the relationship between telomerase activity and telomere binding protein gene expression, MIAPaCa-2 and PANC-1 cells were X-irradiated with a dose of 20 Gy and changes were observed over time. Expression of TRF1 mRNA showed a peak 1 day after irradiation, and then decreased gradually (Figure 7), as did telomerase activity. TRF2 mRNA decreased gradually after X-irradiation (Figure 8), representing a smaller change than for TRF1 showing no correlation with telomerase activity.

### DISCUSSION

Expression of hTR and TEP1 mRNA has been detected not only in cancer cells with high levels of telomerase activity but also in non-neoplastic cells (Nakayama et al, 1998; Ito et al, 1998; Takakura et al, 1998). In contrast, many studies have found expression of hTERT mRNA to be limited to cancer cells (Nakamura et al, 1997; Meyerson et al, 1997; Nakayama et al, 1998; Takakura et al, 1998). In addition, in recent investigations where hTERT and hTR genes were transfected into normal cells, the cells acquired ability to restore telomeres, and the lifespan of the cell culture was extended by population doubling level (Weinrich et al, 1997; Bodnar et al, 1998). Even hTERT transfected by itself could reconstitute telomerase activity (Nakayama et al, 1998; Vaziri et al, 1998). Accordingly, hTERT appears to be the main factor

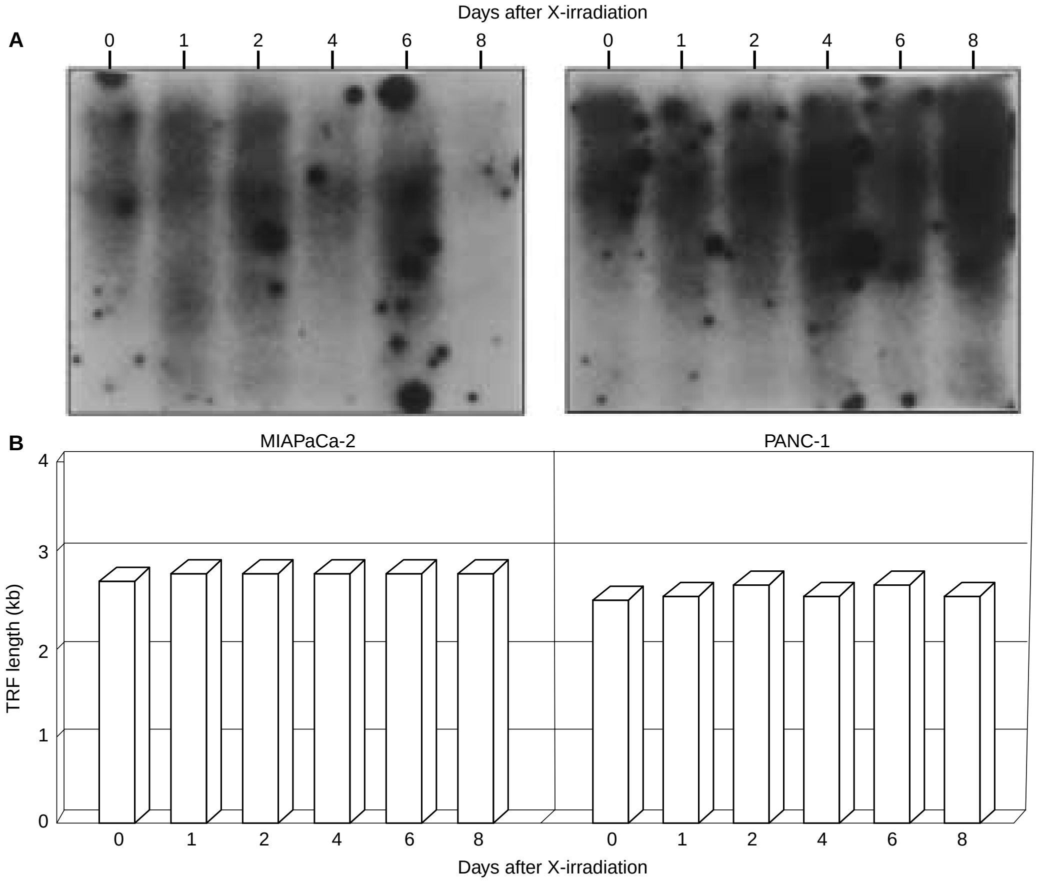

**Figure 6** Telomeric restriction fragment (TRF) length in MIAPaCa-2 and PANC-1 cells before and after X-irradiation. TRF length was determined by the Southern hybridization assay using radioactively labelled TTAGGG probe

BJOC 01-1954 752-757 20/8/01 3:18 pm Page 756

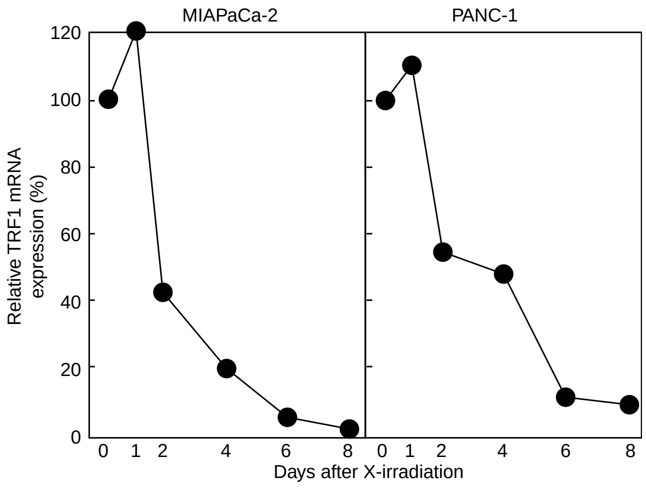

**Figure 7** TRF1 mRNA and TRF2 mRNA expression over time after 20 Gy of X-irradiation to MIAPaCa-2 and PANC-1 cells. Expression of TRF1 mRNA and TRF2 mRNA were measured by a quantitative TaqMan RT-PCR assay, and are stated relative to that of glyceraldehyde-3-phosphate dehydrogenase. Relative TRF1 mRNA and TRF2 mRNA expression is calculated as a relative percentage of cells just before X-irradiation. TRF 1, human telomeric-repeat binding factor protein 1; TRF 2, human telomeric-repeat binding factor protein 2

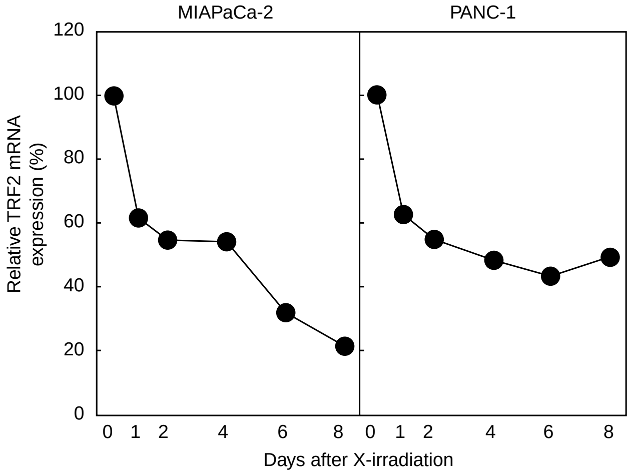

**Figure 8** TRF2 mRNA expression over time after 20 Gy of X-irradiation to MIAPaCa-2 and PANC-1 cells. Expression of TRF2 mRNA was measured by a quantitative TaMan RT-PCR assay, and is stated relative to that of glyceraldehyde-3-phosphate dehydrogenase. Relative TRF2 mRNA expression is calculated as a relative percentage of cells just before X-irradiation. TRF2, human telomeric-repeat binding factor protein 2

permitting regulation of telomerase activity. However, recent studies have detected expression of hTERT mRNA in normal cells (Ito et al, 1998; Kolquist et al, 1998). Precise comparison of expression levels of these telomerase component genes between studies often is difficult because of differences between Northern blotting and RT-PCR methods.

Hyeon et al, have reported increased telomerase activity of human colon carcinoma cell line (SW480) and human nonpolyposis colorectal carcinoma (HNPCC) cell lines (NA50600, NA59 and NA61) was observed at 24 h after 2 or 4 Gy X-irradiation (Hyeon et al, 1998). But, there are no previous reports that showed telomerase activity, telomere length and gene expression of these telomerase components and the telomeric-repeat binding factor proteins in sequential observation following X-irradiation of cultured pancreatic cancer cells. We used X-ray irradiation (10, 20, and 30 Gy) to trigger the telomerase modulating effects upon the pancreatic cells and this is presumably to effect a fixed level of induced damage at the DNA level. In addition, we used low dose X-ray irradiation (1, 3, and 5 Gray) to trigger the telomerase modulating effects, but these doses were too weak to modulate the time-courses of the telomerase expression. Cytotoxic effect of 20 Gy X-irradiation is weaker than that of 1 M Adriamycin (Watanabe et al, 1997).

To examine the telomerase activity and its regulation, we examined the time course of changes in telomerase activity and gene expression for human telomerase components using a quantitative RT-PCR assay with a TaqMan fluorogenic detection system in X-irradiated pancreatic cancer cell lines, finding only hTERT mRNA to parallel telomerase activity. Expression of hTR and TEP1 mRNA increased gradually after X-irradiation, why this occurred are unclear, but these component have a much smaller role to play for telomerase activity, if any. Under all conditions, PANC-1 cells were more resistant to X-irradiation than MIAPaCa-2 cells. But our results found MIAPaCa-2 to parallel PANC-1 in time-course of telomerase activity, expression of its components and telomere length after X-irradiation, and indicate that telomerase and telomere length do not play a role in the differential radiosensitivity of these two cell lines.

More recently, genes encoding TRF1 and TRF2 have been cloned (Chong et al, 1995; Broccoli et al, 1997). TRF1 reportedly inhibits the action of telomerase at the telomeric region (van Steensel et al, 1997), and TRF2 prevents fusion of chromosome ends and, in vitro, to remodel linear telomeric DNA into large duplex loops (van Steensel et al, 1998; Griffith et al, 1999). However, the functional relationship between telomerase activity and gene expression for TRFs in elongation of the telomere has not been clarified. We therefore examined the time course of the telomere length and gene expression for TRFs after irradiation of the cultures. After irradiation, expression of TRF1 decreased when telomerase activity decreased, a change consistent with the function of maintaining a fixed telomere length. This interpretation is in agreement with our data that telomere length was stable in MIAPaCa-2 and PANC-1 cells before and after X-irradiation, and a previous report that cancer cells have a fixed telomere length regardless of division frequency (van Steensel et al, 1997; Counter et al, 1992). TRF1 plays an important role in determining telomere length in cancer cell lines, as TRF1 controls binding of the enzyme to the chromosomal region involved. In this study, TRF2 mRNA decreased gradually after X-irradiation. Hande et al. have reported increased frequencies of dicentricism and translocation along with other types of chromosomal aberrations following X-irradiation (Hyeon et al, 1998; Hande et al, 1998). Fusion of the chromosomal end after X-irradiation might result from such a decrease of TRF2.

Given the correlations observed in cancer cell lines in our study, it is demonstrated that not only hTERT but also TRF1 may play important role of telomerase activity.

### REFERENCES

Bodnar AG, Ouellette M, Frolkis M, Holt SE, Chiu CP, Morin GB, Harley CB, Shay JW, Lichtsteiner S and Wright WE (1998) Extension of life-span by introduction of telomerase into normal human cells. *Science* **279**: 349–352

Broccoli D, Smogorzewska A, Chong L and De Lange T (1997) Human telomeres contain two distinct Myb-related proteins, TRF1 and TRF2. *Nat Genet* **17**: 231–235

 *© 2001 Cancer Research Campaign*

Chong L, van Steensel B, Broccoli D, Erdjument-Bromage H, Hanish J, Tempst P and de Lange T (1995) A human telomeric protein. *Science* **270**: 1663–1667

Counter CM, Avilion AA, Lefeuvre CE, Stewart NG, Greider CW, Harley CB and Bacchetti S (1992) Telomere shortening associated with chromosome instability is arrested in immortal cells which express telomerase activity. *EMBO J* **11**: 1921–1929

Feng J, Funk WD, Wang SS, Weinrich SL, Avilion AA, Chiu CP, Adams RR, Chang E, Allsopp RC, Yu J, LES, West MD, Harley CB, Andrews WH, Greider CW and Villeponteau B (1995) The RNA component of human telomerase. *Science* **269**: 1236–1241

Greider CW and Blackburn EH (1985) Identification of a specific telomere terminal transferase activity in Tetrahymena extracts. *Cell* **43**: 405–413

Griffith JD, Comeau L, Rosenfield S, Stansel RM, Bianchi A, Moss H and de Lange T (1999) Mammalian telomeres end in a large duplex loop. *Cell* **97**: 503–514

Hande MP, Lansdorp PM and Natarajan AT (1998) Induction of telomerase activity by in vivo X-irradiation of mouse splenocytes and its possible role in chromosome healing. *Mutat Res* **404**: 205–214

Harrington L, Mcphail T, Mar V, Zhou W, Oulton R, Program AE, Bass MB, Arruda L and Robinson MO (1997) A mammalian telomerase-associated protein. *Science* **275**: 973–977

Hiyama E, Gollahon L, Kataoka T, Kuroi K, Yokohama T, Gazder AF, Hiyama K, Piatyszek MA and Shay JW (1996) Telomerase activity in human breast cancer. *J Nat Cancer Inst* **88**: 116–122

Hyeon Joo O, Hande MP, Lansdorp PM and Natarajan AT (1998) Induction of telomerase activity and chromosome aberrations in human tumour cell lines following X-irradiation. *Mutat Res* **401**: 121–131

Ito H, Kyo S, Kanaya T, Takamura M, Inoue M and Namiki M (1998) Expression of human telomerase catalytic subunits and correlation with telomerase activity in urotherial cancer. *Clin Cancer Res* **4**: 1603–1608

Kim NW, Piatyszek MA, Prowse KP, Harley CB, West MD, Ho PL, Coviello GM, Wright WE, Weinrich SL and Shay JW (1994) Specific association of human telomerase activity with immortal cells and cancer. *Science* **266**: 2011–2015

Kim NW and Wu F (1997) Advances in quantification and characterization of telomerase activity by the telomeric repeat amplification protocol (TRAP). *Nucleic Acids Res* **25**: 2595–2597

Kolquist KA, Ellisen LW, Counter CM, Meyerson M, Tan LK, Weinberg RA, Haber DA and Gerald WL (1998) Expression of TERT in early premalignant lesions and a subset of cells in normal tissues. *Nat Genet* **19**: 182–186

Kyo S, Kanaya T, Takakura M, Tanaka M, Yamashita A, Inoue H and Inoue M (1999) Expression of human telomerase subunits in ovarian malignant, borderline and benign tumors. *Int J Cancer* **80**: 804–809

Meyerson M, Counter CM, Eaton EN, Ellisen LW, Steiner P, Caddle SD, Ziaugra L, Beijersbergen RL, Davidoff MJ, Liu Q, Bacchetti S, Haber DA and Weinberg, RA (1997) hEST2, the putative human telomerase catalytic subunit gene, is up-regulated in tumor cells and during immortalization. *Cell* **90**: 785–795

Nakamura TM, Morin GB, Chapman KB, Weinrich SL, Andrews WH, Lingner J, Harley CB and Cech TR (1997) Telomerase catalytic subunit homologs from fission yeast and human. *Science* **277**: 955–959

Nakayama J, Saito M, Nakamura H, Matsuura A and Ishikawa F (1997) TLP1: a gene encoding a protein component of mammalian telomerase is a novel member of WD repeats family. *Cell* **88**: 875–884

Nakayama J, Tahara H, Tahara E, Saito M, Ito K, Nakamura H, Nakanishi T, Tahara E, Ide T and Ishikawa F (1998) Telomerase activation by hTRT in human normal fibroblasts and hepatocellular carcinomas. *Nat Genet* **18**: 65–68

Shay JW and Bacchetti S (1997) A survey of telomerase activity in human cancer. *Eur J Cancer* **33**: 787–791

Takakura M, Kyo S, Kanaya T, Tanaka M and Inoue M (1998) Expression of human telomerase subunits and correlation with cervical cancer. *Cancer Res* **58**: 1558–1561

van Steensel B and de Lange T (1997) Control of telomere length by the human telomeric protein TRF1. *Nature* **385**: 740–743

van Steensel B, Smogorzewska A and de Lange T (1998) TRF2 protects human telomeres from end-to-end fusions. *Cell* **92**: 401–413

Vaziri H and Benchimol S (1998) Reconstitution of telomerase activity in normal human cells leads to elongation of telomeres and extended replicative life span. *Curr Biol* **8**: 279–282

Watanabe N, Tsuji N, Tsuji Y, Sasaki H, Okamoto T, Akiyama S, Kobayashi D, Sato T, Yamauchi N and Niitsu Y (1996) Endogenous tumor necrosis factor inhibits the cytotoxicity of exogenous tumor necrosis factor and adriamycin in pancreatic carcinoma cells. *Pancreas* **13**: 395–400

Watanabe N, Tsuji N, Kobayashi D, Yamauchi N, Akiyama S, Sasaki H, Sato T, Okamoto T and Niitsu Y (1997) Endogenous tumor necrosis factor functions as a resistant factor against hyperthermic cytotoxicity in pancreatic carcinoma cells via enhancement of the heart shock element-binding activity of heart shock factor 1. *Chemotherapy* **43**: 406–414

Weinrich SL, Pruzan R, Ma L, Ouellette M, Tesmer VM, Holt SE, Bodnar AG, Lichtsteiner S, Kim NW, TRAGER JB, Taylor RD, Carlos R, Andrews WH, Wright WE, Shay JW, Harley CB and Morin GB (1997) Reconstitution of human telomerase with the template RNA component hTR and the catalytic protein subunit hTRT. *Nat Genet* **17**: 498–502

Yajima T, Yagihashi A, Kameshima H, Kobayashi D, Furuya D, Hirata K and Watanabe N (1998) Quantitative reverse transcription-PCR assay of the RNA component of human telomerase using the TaqMan fluorogenic detection system. *Clin Chem* **44**: 2441–2445

Yajima T, Yagihashi A, Kameshima H, Furuya D, Kobayashi D, Hirata K and Watanabe N (2000) Establishment of quantitative reverse transcription-polymerase chain reaction assays for human telomerase-associated genes. *Clin Chim Acta* **290**: 117–127

*© 2001 Cancer Research Campaign*
